# Increased waist circumference is independently associated with hypothyroidism in Mexican Americans: replicative evidence from two large, population-based studies

**DOI:** 10.1186/1472-6823-14-46

**Published:** 2014-06-10

**Authors:** Manju Mamtani, Hemant Kulkarni, Thomas D Dyer, Laura Almasy, Michael C Mahaney, Ravindranath Duggirala, Anthony G Comuzzie, Paul B Samollow, John Blangero, Joanne E Curran

**Affiliations:** 1 Department of Genetics, Texas Biomedical Research Institute, 7620 NW Loop 410, San Antonio, TX, USA; 2 Department of Veterinary Integrative Biosciences, College of Veterinary Medicine and Biomedical Sciences, Texas A&M University, College Station, TX, USA

**Keywords:** Waist circumference, Central obesity, Thyroid dysfunction, Mexican Americans

## Abstract

**Background:**

Mexican Americans are at an increased risk of both thyroid dysfunction and metabolic syndrome (MS). Thus it is conceivable that some components of the MS may be associated with the risk of thyroid dysfunction in these individuals. Our objective was to investigate and replicate the potential association of MS traits with thyroid dysfunction in Mexican Americans.

**Methods:**

We conducted association testing for 18 MS traits in two large studies on Mexican Americans – the San Antonio Family Heart Study (SAFHS) and the National Health and Nutrition Examination Survey (NHANES) 2007–10. A total of 907 participants from 42 families in SAFHS and 1633 unrelated participants from NHANES 2007–10 were included in this study. The outcome measures were prevalence of clinical and subclinical hypothyroidism and thyroid function index (TFI) – a measure of thyroid function. For the SAFHS, we used polygenic regression analyses with multiple covariates to test associations in setting of family studies. For the NHANES 2007–10, we corrected for the survey design variables as needed for association analyses in survey data. In both datasets, we corrected for age, sex and their linear and quadratic interactions.

**Results:**

TFI was an accurate indicator of clinical thyroid status (area under the receiver-operating-characteristic curve to detect clinical hypothyroidism, 0.98) in both SAFHS and NHANES 2007–10. Of the 18 MS traits, waist circumference (WC) showed the most consistent association with TFI in both studies independently of age, sex and body mass index (BMI). In the SAFHS and NHANES 2007–10 datasets, each standard deviation increase in WC was associated with 0.13 (p < 0.001) and 0.11 (p < 0.001) unit increase in the TFI, respectively. In a series of polygenic and linear regression models, central obesity (defined as WC ≥ 102 cm in men and ≥88 cm in women) was associated with clinical and subclinical hypothyroidism independent of age, sex, BMI and type 2 diabetes in both datasets. Estimated prevalence of hypothyroidism was consistently high in those with central obesity, especially below 45y of age.

**Conclusions:**

WC independently associates with increased risk of thyroid dysfunction. Use of WC to identify Mexican American subjects at high risk of thyroid dysfunction should be investigated in future studies.

## Background

Reportedly 5-10% of the United States population has subclinical or clinical hypothyroidism [1,2]. It is of clinical interest that differential prevalence estimates of hypothyroidism are influenced by age [3], sex , ethnicity [4] and other risk factors like presence of type 2 diabetes (T2D) [5,6]. Further, Mexican American and white ethnicity is associated with a high risk of thyroid dysfunction [3,7-9]. Knowledge of these risk factors is crucial to identify hypothyroidism in its nascent, subclinical stage since it is associated with preventable but dangerous complications like hyperlipidemia, insulin resistance, atherosclerosis and additional risks to mothers and infants [10]. The accuracy of traditional strategies to detect thyroid dysfunction that use a constellation of symptoms and signs has been greatly increased by recent improvements in the assays for thyroxine and thyroid stimulating hormone (TSH). For example, Helfand and Crapo [10] have described that the sensitivity and specificity of TSH to confirm thyroid disease is 98% and 92%, respectively. In primary care settings however, the observed accuracy is not as high. For example, the positive predictive value of a low TSH to detect hyperthyroidism has been reported to be only 24% while that of a high TSH to detect hypothyroidism is only 6% [10]. Improved screening programs for thyroid dysfunction will therefore need detection of differential thyroid dysfunction risks across epidemiologically diverse groups.

There is now burgeoning evidence that vindicates a potential link between thyroid dysfunction and metabolic syndrome (MS). Even in a euthyroid state, a high normal TSH is strongly associated with MS [11]. Similarly, in the National Health and Nutrition Examination Survey (NHANES) 2007–2008 data [4], body mass index (BMI) and waist circumference (WC) were associated with serum TSH levels. More recently, the Health, Ageing and Body Composition Study [12] reported that unit increase in TSH was associated with a 3% increase in the odds of MS. It is also instructive in this regard that mutations in the thyroid hormone receptor beta (THRB) gene are associated with increased energy intake, hyperphagia and resting energy expenditure [13]. It is noteworthy, however, that MS represents a constellation of correlated phenotypic traits that together capture a wide spectrum of metabolic disorders including prediabetes, type 2 diabetes, insulin resistance, hypertension, obesity and dyslipidemia [14]. However, the relative and comparative contribution of these individual components of MS to thyroid dysfunction is currently unknown.

Since Mexican Americans are at an increased risk of MS, here we investigated the associations of MS-related traits with indicators of thyroid dysfunction in Mexican Americans from two large studies – San Antonio Family Heart Study (SAFHS) [15,16] and NHANES 2007–10. The former study is uniquely suited to investigate the hypothesized association between MS and thyroid function in families who are at a high risk of both MS and thyroid abnormalities while the latter study provides a rich, nationally representative population based setting. We aimed to investigate and replicate the MS-thyroid function nexus in these epidemiologically distinct scenarios using an appropriate and robust analytical approach. Here we report our finding that central obesity – a component of MS – is additively, independently and significantly associated with altered thyroid function.

## Methods

### Study participants

#### San Antonio Family Heart Study (SAFHS)

The recruitment and ascertainment procedures used in the SAFHS have been described in details elsewhere [15-17]. Briefly, the study has now recruited over 1600 subjects from 42 large and extended families with a majority of these subjects having completed up to three additional follow-up visits spaced ~5 years apart. In the analyses presented here we used the data and samples collected during the first visit. Profiling of thyroid-related traits was available for 919 subjects (from 42 families). Informed consent was obtained from all participants before collection of samples. The Institutional Review Board of the University of Texas Health Sciences Center at San Antonio approved the study.

#### The National Health and Nutrition Examination Survey (NHANES) 2007–10

NHANES is a yearly survey conducted by the National Center of Health Statistics of the Centers for Disease Control and Prevention (CDC). These datasets are publicly available in a de-identified fashion. Detailed description of the NHANES 2007–10 survey and sampling strategies can be found online at http://www.cdc.gov/nchs/nhanes.htm. The NHANES 2007–10 dataset contained thyroid related components. From this multi-ethnic dataset, we selected Mexican American responders (n = 1636). Detailed characteristics of the SAFHS and NHANES 2007–10 participants are shown in Table 1.

**Table 1:** Characteristics of the study subjects

**Characteristic**	**SAFHS (n = 919)**		**NHANES 2007–10 (n = 1636)**	
	**Value**	**N**	**Value**	**N**
**Demographics**				
Age [mean (SE)] y	38.92 (0.54)	907	35.42 (0.81^†^)	1636
Females [n (%)]	555 (61.19)	907	842 (47.98^††^)	1636
Diabetes at enrolment [n (%)]	126 (13.91)	906	170 (7.35)	1635
**Anthropometric indexes** [mean (SE)]				
Waist cm	94.71 (0.58)	898	95.65 (0.68)	1589
BMI Kg/m^2^	29.50 (0.23)	900	28.49 (0.27)	1618
WHR	0.89 (0.003)	897	-	-
**Blood pressure** [mean (SE)]				
Systolic mmHg	119.96 (0.61)	899	116.98 (0.75)	1504
Diastolic mmHg	70.56 (0.34)	899	66.84 (0.56)	1504
**Biochemical indexes** [mean (SE)]				
Fasting glucose mmol/L	5.59 (0.07)	907	6.00 (0.11)	805
Fasting insulin μU/mL	16.72 (0.65)	894	15.64 (0.63)	803
Total serum cholesterol mg/dl	189.06 (1.32)	906	190.35 (1.54)	1636
Serum triglycerides mg/dl	151.56 (4.40)	906	141.15 (3.31)	805
HDL cholesterol mg/dl	50.54 (0.43)	905	48.90 (0.31)	1636
LDL cholesterol mg/dl	109.92 (1.10)	906	112.42 (1.52)	785
**Thyroid related traits** [mean (SE)]				
Total thyroxine μg/dl	7.63 (0.07)	839	8.19 (0.08)	1628
Free thyroxine ng/dl	1.38 (0.01)	907	0.80 (0.01)	1636
Total triiodothyronine ng/dl	122.15 (0.92)	845	122.89 (1.03)	1635
Free triiodothyronine pg/ml	3.62 (0.04)	907	3.42 (0.03)	1634
Reverse triiodothyronine ng/dl	23.00 (0.30)	451	-	-
Thyroxine binding globulin μg/ml	19.84 (0.19)	856	-	-
Thyroglobulin ng/ml	11.56 (1.11)	879	11.27 (1.15)	1635
Thyroid stimulating hormone μU/ml	2.96 (0.14)	907	2.00 (0.14)	1635

^†^All proportions in NHANES samples are adjusted for survey design variables.

^††^For NHANES samples standard errors represent linearized standard errors.

### Phenotypic traits related to thyroid function

#### Traits in SAFHS and NHANES 2007–10

In the SAFHS, available thyroid-related traits were total and free thyroxine (TT4 and FT4), total and free triiodothyronine (TT3 and FT3), reverse triiodothyronine (RT3C), thyroxine binding globulin (TBG), thyroglobulin (THG) and thyroid stimulating hormone (TSH). R3TC was available for only 451 (49.1%) subjects. The experimental methods used for measuring thyroid-related traits in the SAFHS have been described previously [18]. Of these traits, data on R3TC and TBG was not available in the NHANES 2007–10 dataset. Thyroid profile data for both studies is shown in Table 1. Finally, to capture the information content of TSH, FT3 and FT4 in a single, composite and continuous measure we developed an index [which we termed the thyroid function index (TFI)] as: TFI = TSH/(FT3 × FT4). We conceptualized this index on the basis of the clinical expectation that high values of TSH combined with low values of FT3 and FT4 values would be indicative of hypothyroidism and, conversely, low value of TSH combined with high values of FT3 and FT4 would be indicative of hyperthyroidism. Before conducting any association analyses for the TFI trait, we first validated this index in both SAFHS and NHANES 2007–10 participants.

#### Clinical thyroid status

Based on the values of TSH, FT4 and FT3, we used the recommended definitions [19,20] of thyroid status as follows: clinical hypothyroidism (CO) – TSH ≥3.0 μIU/ml AND (FT3 < 3.5 pmol/L OR FT4 < 10 pmol/L); subclinical hypothyroidism (SO) – TSH ≥3.0 μIU/ml AND (FT3 ≥ 3.5 pmol/L OR FT4 ≥ 10 pmol/L); clinical hyperthyroidism (CR) – TSH <0.3 μIU/ml AND (FT3 ≥ 6.5 pmol/L OR FT4 ≥ 19 pmol/L); subclinical hyperthyroidism (SR) – TSH <0.3 μIU/ml AND (FT3 < 6.5 pmol/L OR FT4 < 19 pmol/L). Further, we defined hypothyroidism as presence of clinical or subclinical hypothyroidism.

### Metabolic syndrome related traits

We used data on 18 phenotypic traits related to metabolic syndrome (12 continuous and six dichotomous). The continuous traits included fasting and post-glucose load plasma levels of glucose and insulin (measured by 2-hour oral glucose tolerance test); two homeostasis model of assessment (HOMA) measures – HOMA-IR representing insulin resistance and HOMA-β representing β-cell function; three measures of obesity – body mass index (BMI), WC and waist-hip ratio (WHR); systolic and diastolic blood pressure; and three serum lipid measures – total cholesterol, triglycerides and high-density lipoprotein cholesterol (HDL-C). The methods of assessment for these traits in the SAFHS and NHANES 2007–10 participants have been extensively described. ( [16,17,21] and http://wwwn.cdc.gov/nchs/nhanes/search/ nhanes07_08.aspx/analyticnote_2007-2010.pdf. The definitions of the six dichotomous traits (central obesity, raised triglycerides, low HDL-C, T2D, high blood pressure and metabolic syndrome) are provided in Additional file 1: Table S1.

### Statistical analyses

#### Inverse normalization of continuous traits

To ensure that i) inferences regarding association were not influenced by a potentially non-normal distribution of the continuous traits and ii) the results from the SAFHS and NHANES 2007–10 can be evaluated using a comparative metric, we inverse-normalized all continuous traits before conducting regression analyses. This transformation was used to generate inverse-normalized traits having a mean of 0 and a standard deviation of 1 which was then used in the regression analyses.

#### Validation of TFI

We validated the TFI using receiver operating characteristic (ROC) curves and estimating the comparative area under the ROC curve (AUC) for the individual components of TFI and TFI *per se*. To estimate AUC, we used the Wilcoxon statistic and its standard error as described by Hanley and McNeil [22]. We used the STATA 12.0 package and roctab program to estimate these statistics.

#### Polygenic regression analyses in SAFHS

We examined association between TFI and phenotypic traits related to MS in the SAFHS participants using polygenic regression models in a variance-components framework [23,24]. These models implicitly account for the genetic correlations and kinship structures and do not consider the individual subjects as an independent unit. The polygenic regression models were of the following form: *TFI*_*i*_ = *m* + ∑ *b*_*k*_*a*_*ik*_ + *g*_*i*_ + *e*_*i*_ where, TFI is the inverse-normalized log-transformed thyroid function index; m is the trait mean; a is the covariate vector of dimension k with b as the corresponding regression coefficients; g is the polygenic effect and e is the residual error for an individual indexed by i. The term g was modeled as random effects based on the coefficients of relationship in the kinship matrix. All models were first run with only the MS-related trait as the covariate (unadjusted models) and then additionally included adjustments for age, age^2^, sex, age x sex and age^2^ x sex interactions as covariates (adjusted models). Further, we adjusted these models for the use of lipid lowering, antihypertensive and antidiabetic agents. Statistical significance of the association between a MS-related trait and TFI was tested by constraining the corresponding regression coefficient to zero and comparing the log-likelihoods of the constrained and unconstrained models in a likelihood ratio χ^2^ test. These analyses were conducted using the SOLAR software package [25]. Statistical significance was tested at a global type I error rate of 0.05.

#### Survey design-corrected regression analyses in NHANES 2007–10 dataset

Since the NHANES dataset is based on a complex sampling strategy to recruit a nationally representative sample, we used appropriate survey based analytical methods as detailed on the Centers for Disease Control and Prevention website (http://www.cdc.gov/nchs/tutorials/nhanes/). Briefly, we corrected for the proportional sampling units and the sampling weights using the svyset command in STATA 12.0 (Stata Corp, College Station, TX) software package. Since it was likely that the subset of Mexican Americans included in this study could have come from some specific sampling units, we used the singleunit(scaled) option in the svyset command. All regression analyses were then conducted using the svy command and regress subcommand.

#### Correction for multiple testing

We used 18 MS traits that are conceptually correlated with each other to varying degrees. Therefore, these traits do not represent independent tests. To estimate the effective number of independent tests we used the method of Li and Ji [26] which relies upon the eigenvalues of the full correlation matrix for the traits and then uses a Sidak type correction to estimate the corrected type I error rate for a global alpha error of 0.05. Using this method we estimated that the corrected alpha for the SAFHS dataset was 0.0051 and for the NHANES dataset it was 0.0073.

#### Prevalence of hypothyroidism

In the SAFHS participants, we used a liability threshold model approach to determine the prevalence of hypothyroidism within combinatorial classes determined by age categories and presence or absence of central obesity. The polygenic regression models used in this scenario were specifically tailored for discrete traits and additively accounted for kinship structure. In the NHANES 2007–10 participants, we estimated the proportion of hypothyroidism within each class after correcting for the survey design variables (sampling weights, proportionality sampling units and strata) using the svy command in STATA software package.

## Results

### Study participants

**Figure 1:**
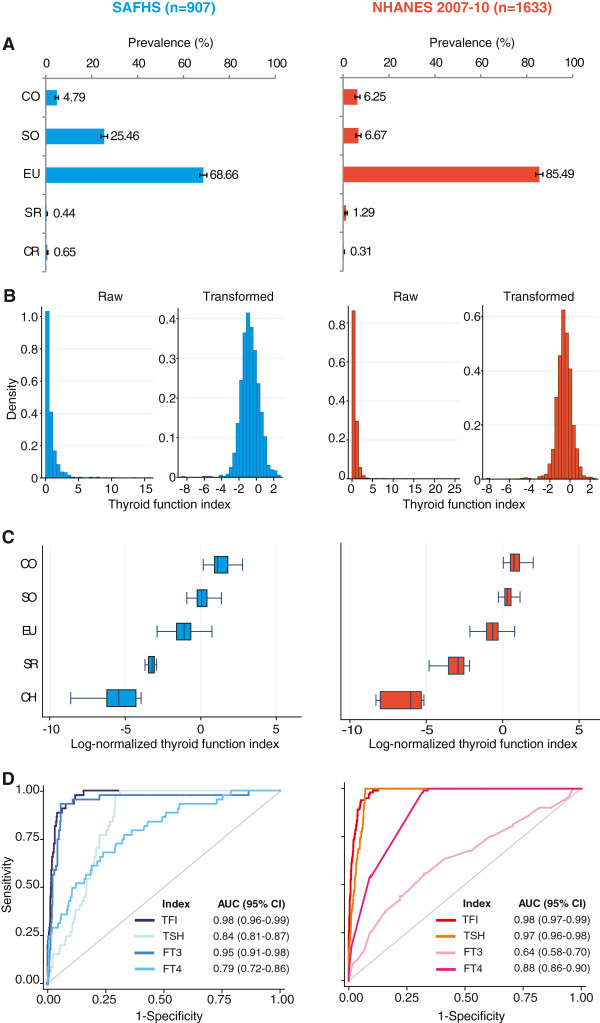
**Development and validation of thyroid function index (TFI) as a marker of thyroid function.** Results are color coded as blue for SAFHS and red for NHANES 2007–10 participants. **(A)** Spectrum of clinical thyroid status. Bars represent prevalence (%) while error bars represent 95% confidence interval. CO, clinical hypothyroidism; SO, subclinical hypothyroidism; EU, euthyroid; SR, subclinical hyperthyroidism; CR, clinical hyperthyroidism. **(B)** Effect of log-transformation on distribution of TFI. Left and right panels show histograms for raw and log-transformed TFI, respectively. **(C)** Box and whisker plots showing distribution of log-normalized TFI by thyroid status. **(D)** Receiver operating characteristics (ROC) curves comparing the discriminatory performance of TFI, TSH, FT3 and FT4 to detect clinical hypothyroidism. AUC, area under the ROC curve; CI, confidence interval.

Of the 919 SAFHS and 1636 NHANES 2007–10 participants who underwent thyroid profiling, we included 907 SAFHS and 1633 NHANES 2007–10 participants for whom data on FT3, FT4 and TSH were available. We observed (Table 1) that the SAFHS participants were slightly older (~3 y), had a higher proportion of females (~12%) and had a higher prevalence of diabetes (~2 times) as compared to the NHANES 2007–10 responders. However, the anthropometric indexes, blood pressure, oral glucose tolerance test results and serum lipid profiles were similar in both studies. With regard to the thyroid profile, the mean FT4 and TSH levels were higher in SAFHS participants but other thyroid-related traits were comparable across the two studies. Consequently, the prevalence of clinical hypothyroidism was comparable across the two studies but the prevalence of subclinical hypothyroidism was higher in the SAFHS participants than the NHANES responders (25.5% vs 6.7%, Figure 1A). For the SAFHS, informed consent was obtained from all participants before data and sample collection. The study was approved by the Institutional Review Board of the University of Texas Health Science Center at San Antonio. The NHANES study has been approved by the National Center of Health Statistics Institutional Review Board under protocols #98-12 and #2005-06. The study involved interviews, collection of biological samples after taking informed consent from all participants. All protocols used in the study are in accordance with the Declaration of Helsinki.

### Validation of thyroid function index

The distribution of TFI was heavily skewed in both studies (Figure 1B, left panels). Therefore, we log-transformed the TFI and found that the log-transformed TFI was distributed symmetrically in both datasets (Figure 1B, right panels). However, we observed that the log-transformed TFI had a non-significant skew in the SAFHS dataset (p = 0.168) but not in the NHANES 2007–10 dataset (p < 0.001). Therefore, in all ensuing analyses we further corrected for this departure from normality by using an inverse-normalization method. From this point forward, TFI implies inverse-normalized, log-transformed TFI.The TFI demonstrated (Figure 1C) a clear gradient of distribution with very low values corroborating clinical hyperthyroidism and high values characterizing clinical hypothyroidism. In a head to head comparative evaluation of TFI and its individual components to diagnose clinical hypothyroidism (Figure 1D), we observed that the TFI superseded all of its individual components in both studies. Specifically, the AUC for TFI was 98% while the AUCs for TSH, FT3 and FT4 were consistently below that of TFI in both the studies. These results therefore validated the potential use of TFI as a replicable and generalizable continuous measure of thyroid function.

### MS-related traits and TFI: SAFHS participants

We studied the association of 18 MS-related traits with TFI in SAFHS participants after accounting for the kinship structure using a series of polygenic regression models (Table 2). Even after accounting for age, sex and their interactions in addition to the kinship structure, we found that WC (β = 0.13, p = 0.0002), BMI (β = 0.11, p = 0.0015), WHR (β = 0.09, p = 0.0103) and serum triglycerides (β = 0.08, p = 0.0183) were significantly associated with TFI. Consequently, central obesity (defined on the basis of WC) and metabolic syndrome were also significantly associated with TFI. In males, WHR (β = 0.20, p = 0.008) and metabolic syndrome (β = 0.25, p = 0.0379) were the only two traits significantly associated with TFI. However, in females WC (β = 0.14, p = 0.001), BMI (β = 0.13, p = 0.0026), WHR (β = 0.10, p = 0.022) and central obesity (β = 0.20, p = 0.0247) achieved statistical significance for association with TFI. Thus, these results indicated a consistent association of central obesity with TFI in the SAFHS participants.

**Table 2:** Association of metabolic syndrome related traits with thyroid function index in the SAFHS participants

**Metabolic syndrome**-**related trait**	**All subjects**				**Males**				**Females**			
	**Unadjusted**		**Adjusted**^**†**^		**Unadjusted**		**Adjusted**^**††**^		**Unadjusted**		**Adjusted**^**††**^	
	**β**	**p**	**β**	**p**	**β**	**p**	**β**	**p**	**β**	**P**	**β**	**p**
Fasting glucose	0.08	0.03	-0.02	0.62	0.07	0.19	0.01	0.91	0.07	0.14	-0.03	0.53
2-hour glucose	0.14	<0.0001	0.05	0.15	0.16	<0.01	0.11	0.05	0.09	0.06	-0.01	0.82
Fasting insulin	0.04	0.27	0.00	0.96	0.03	0.62	0.01	0.78	0.03	0.52	-0.01	0.77
2-hour insulin	0.08	0.02	0.04	0.22	0.07	0.18	0.04	0.43	0.07	0.13	0.03	0.47
HOMA-IR	0.06	0.12	0.00	0.94	0.04	0.41	0.02	0.78	0.05	0.33	-0.02	0.68
HOMA-β	-0.07	0.06	0.02	0.58	-0.05	0.38	0.01	0.86	-0.06	0.1763	0.04	0.47
WC	0.19	<0.0001	**0.13**	**<0.001**	0.12	0.04	0.08	0.18	0.20	<0.0001	**0.14**	**0.001**
BMI	0.15	<0.0001	**0.11**	**<0.01**	0.04	0.45	0.02	0.76	0.18	<0.0001	**0.13**	**<0.01**
WHR	0.15	<0.0001	0.09	0.01	0.23	<0.001	0.20	<0.01	0.16	0.0001	0.10	0.02
Total serum cholesterol	0.12	0.0001	0.06	0.06	0.08	0.10	0.07	0.19	0.11	0.0101	0.03	0.46
Serum triglycerides	0.15	<0.0001	0.08	0.02	0.12	0.02	0.09	0.07	0.14	<0.01	0.07	0.14
Serum HDL cholesterol	0.05	0.11	0.06	0.06	0.05	0.32	0.07	0.15	0.02	0.67	0.02	0.69
Central obesity	0.30	<0.0001	**0.20**	**<0.01**	0.16	0.14	0.13	0.26	0.33	0.0001	0.20	0.02
Raised triglycerides	0.16	0.02	0.05	0.42	0.13	0.19	0.09	0.39	0.19	0.03	0.06	0.49
Low HDL cholesterol	-0.02	0.81	-0.05	0.46	0.12	0.28	0.08	0.49	-0.11	0.20	-0.11	0.17
High blood pressure	-0.25	<0.01	-0.05	0.61	-0.10	0.44	0.06	0.65	-0.26	0.01	-0.06	0.61
Type 2 diabetes	0.15	0.04	-0.04	0.60	0.14	0.22	-0.01	0.95	0.15	0.14	-0.04	0.70
Metabolic syndrome	0.27	<0.0001	0.16	0.02	0.28	0.02	0.25	0.04	0.24	<0.01	0.11	0.24

^†^Adjusted for age, age^2^, sex, age x sex interaction and age^2^ x sex interaction; ^††^, adjusted for age and age^2^; numbers in bold face indicate statistically significant associations after correcting for multiple testing.

### MS-related traits and TFI: NHANES 2007–10

We conducted conceptually similar analyses in the NHANES 2007–10 participants. Here we used the appropriate survey design correction methods to statistically account for the potential confounding due to study design. We observed (Table 3) that after correcting for age, sex and their interactions, HOMA-IR (β = 0.06, p = 0.0448), WC (β = 0.11, p = 0.0008), BMI (β = 0.12, p = 0.0004), total serum cholesterol (β = 0.11, p = 0.0014) and serum triglycerides (β = 0.09, p = 0.036) were statistically significantly associated with TFI. In males, several traits were significantly associated with TFI but the strongest associations were again observed for WC (β = 0.20, p = 7.6 × 10^-8^), BMI (β = 0.20, p = 1.3 × 10^-7^) and central obesity (β = 0.30, p = 5.0 × 10^-5^). However, in females only total serum cholesterol (β = 0.11, p = 0.0437) was statistically significantly associated.

**Table 3:** **Association of metabolic syndrome related traits with thyroid function index in NHANES 2007**–2010 participants*

**Metabolic syndrome**-**related trait**	**All subjects**				**Males**				**Females**			
	**Unadjusted**		**Adjusted**^**†**^		**Unadjusted**		**Adjusted**^**††**^		**Unadjusted**		**Adjusted**^**††**^	
	**β**	**p**	**β**	**P**	**β**	**p**	**β**	**p**	**β**	**P**	**β**	**P**
Fasting glucose	0.09	0.08	0.01	0.78	0.15	<0.01	0.12	0.03	0.08	0.25	-0.02	0.76
Fasting insulin	0.05	0.13	0.06	0.07	0.12	<0.01	0.12	<0.01	-0.05	0.32	-0.03	0.56
HOMA-IR	0.07	0.02	0.06	0.04	0.15	<0.001	0.14	<0.001	-0.02	0.70	-0.03	0.52
HOMA-β	0.00	0.98	0.05	0.20	0.05	0.30	0.08	0.12	-0.09	0.16	0.00	0.98
WC	0.16	<0.0001	**0.11**	**<0.001**	0.21	<0.0001	0.20	**<0.0001**	0.13	<0.001	0.06	0.17
BMI	0.15	<0.0001	**0.12**	**<0.001**	0.20	<0.0001	0.20	**<0.0001**	0.11	<0.01	0.03	0.40
Total serum cholesterol	0.16	<0.0001	**0.11**	**<0.01**	0.13	<0.001	0.13	**<0.001**	0.20	<0.001	0.11	0.04
Serum triglycerides	0.13	<0.001	0.09	0.04	0.11	<0.01	0.10	0.02	0.20	0.001	0.12	0.07
Serum HDL cholesterol	0.00	0.98	0.00	0.85	-0.10	<0.001	-0.10	<0.001	0.05	0.09	0.04	0.11
Central obesity	0.32	<0.0001	**0.22**	**<0.001**	0.36	<0.0001	0.30	**<0.0001**	0.22	<0.01	0.05	0.58
Raised triglycerides	0.17	<0.01	0.01	0.90	0.09	0.17	-0.01	0.88	0.31	<0.01	0.13	0.20
Low HDL cholesterol	0.10	<0.01	0.04	0.12	0.14	0.02	0.10	0.08	0.02	0.69	-0.03	0.52
High blood pressure	0.31	<0.0001	0.08	0.16	0.32	<0.01	0.17	0.13	0.34	<0.001	0.02	0.83
Type 2 diabetes	0.30	<0.0001	0.04	0.55	0.24	0.02	0.00	0.98	0.33	0.001	0.07	0.47
Metabolic syndrome	0.32	<0.0001	0.16	0.05	0.29	0.01	0.19	0.11	0.29	0.06	0.09	0.62

*In this set, data on 2-hour glucose, 2-hour insulin and WHR were not available; ^†^, adjusted for age, age^2^, sex, age x sex interaction and age^2^ x sex interaction; ^††^, adjusted for age and age^2^; numbers in bold face indicate statistically significant associations after correcting for multiple testing.

### Association of WC with TFI is independent of BMI

Since both WC and BMI were positively associated with TFI in both studies, we compared the strength of association of these two traits with TFI using interactive models (Additional file 1: Table S2). Our results indicated that in both the studies WC was significantly associated with TFI whether analyzed separately in males (β = 0.40, p = 0.001 in SAFHS and β = 0.28, p = 0.004 in NHANES 2007–10) and females (β = 0.19, p = 0.018 in SAFHS and β = 0.19, p = 0.07 in NHANES 2007–10) or in all participants together (β = 0.22, p = 0.001 in SAFHS and β = 0.13, p = 0.066 in NHANES 2007–10). Interestingly, a stronger association signal between WC and TFI was observed in males as compared to females in both SAFHS and NAHNES 2007–10 participants in interactive models. These results together indicated that the association of WC with TFI is independent of the potential confounding due to BMI.

Since it is clinically more appealing to use cutoffs of WC and BMI, we used the similar approach to test whether central obesity (defined on the basis of WC) is associated with TFI independent of general obesity (defined as BMI ≥ 30 kg/m^2^). The results of these analyses are shown in Additional file 1: Table S3 and corroborate the inferences drawn from results in Additional file 1: Table S2. Therefore, to facilitate clinical use we evaluated central obesity (instead of WC) as an independent determinant of thyroid dysfunction in the succeeding analyses.

### Central obesity associates with TFI independently of age

**Figure 2:**
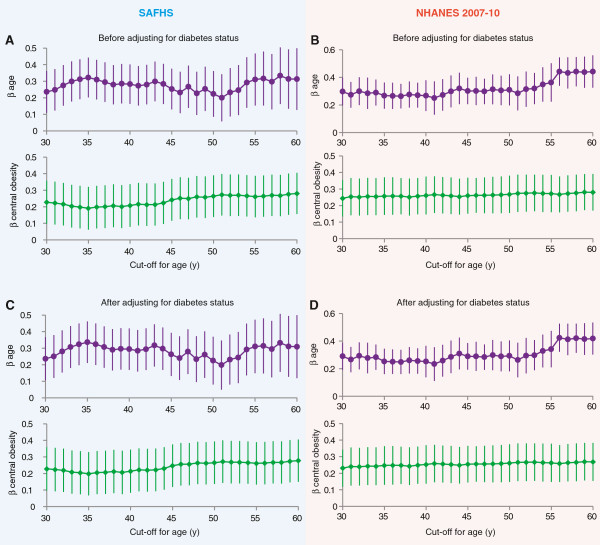
**Association of central obesity with TFI is independent of age.** Results for SAFHS participants are shown on the blue background on left while those for the NHANES 2007–10 participants are shown on pink background on right. **(A-B)** Estimated regression coefficients (β) for age (purple circles) and central obesity (green diamonds) in a single polygenic model for each cutoff of age. In these analyses age was dichotomized at the cutoffs shown on the abscissa and polygenic regression was run at each cutoff. Error bars represent 95% confidence intervals around the regression coefficient. **(C-D)** Analyses similar to those in **(A-B)** but additionally accounting for presence of type 2 diabetes.

Since central obesity and thyroid dysfunction are both associated with age, we next investigated whether the association of central obesity with TFI is independent of age. For this, we slid the age cutoff over the range 30-60 y and at each cutoff ran a polygenic regression model that included dichotomized age and central obesity as covariates and TFI as the dependent variable. We observed that in SAFHS as well as NHANES 2007–10 participants, the estimated regression coefficient for central obesity (green diamonds and bars in Figure 2A and 2B) was robustly stable and hovered between 0.2 and 0.3 irrespective of the cutoff for age. Further, as existing T2D is sometimes considered an indication for screening of thyroid dysfunction, we adjusted these models for presence of T2D also. We observed (Figure 2C and 2D) that the significant and independent association of central obesity with TFI was sustained even after accounting for age and presence of T2D.

### Prevalence of hypothyroidism based on central obesity and age

**Figure 3:**
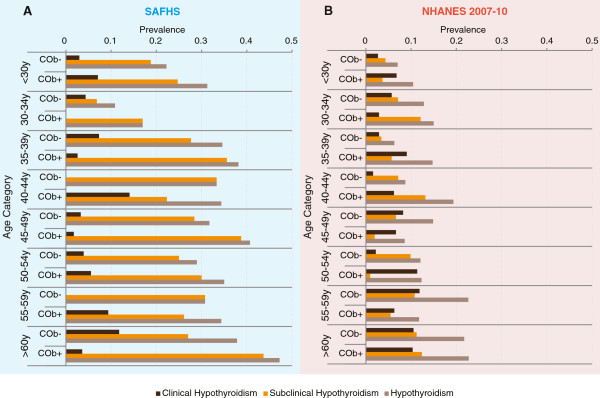
**Utility of central obesity as an additional screening measure to detect thyroid dysfunction.** Results for SAFHS participants are shown on the blue background in **(A)** while those for the NHANES 2007–10 participants are shown on pink background in **(B)**. Each panel shows color coded bars representing prevalence of the indicated clinical thyroid status within each age category. COb+, central obesity present; COb-, central obesity absent.

Lastly, we estimated prevalence of clinical and subclinical hypothyroidism in subgroups defined by 5-yearly age intervals and presence or absence of central obesity. In SAFHS participants (Figure 3A), prevalence of hypothyroidism was higher within each age subgroup in those with central obesity than without. In the NHANES 2007–10 participants (Figure 3B), this finding was replicated in all age categories except 45–49 and 55-59 y. Thus, central obesity showed an additive and independent screening benefit especially below the age of 45 y.

## Discussion

Our results demonstrate that WC, as a measure of central obesity, is a significant and independent indicator of thyroid dysfunction in Mexican Americans. These results are substantiated by the fact that the associative patterns were consistently observed in two large and disparate studies on the same ethnic population. Interestingly, the prevalence of clinical and subclinical hypothyroidism was higher in the SAFHS than that in the NHANES 2007–10 participants. This finding is important because subjects with subclinical hypothyroidism are at a higher risk of eventual overt hypothyroidism in future as compared to the euthyroid subjects [27] and our results imply that such subjects may be concentrated within families. Our findings therefore indicate that central obesity may be not only an important player in the multifactorial web of thyroid dysfunction but may also contribute to the development of future clinical hypothyroidism by predicting the subjects who are currently at a high risk of subclinical hypothyroidism.

The prevalence of subclinical and clinical hypothyroidism estimated in this study may be somewhat higher than its true population value for two reasons. First, there is substantial evidence in the literature supporting the view that TSH values may be increased in obesity and morbid obesity [28-31]. Second, the TSH cutoff used to define hypothyroidism here is restrictive. It is not uncommon to use 5 μU/ml as the upper limit of normal TSH [19,32,33]. If this high cutoff value is used then expectedly the prevalence of hypothyroidism will be less than that reported here. We chose to use the cutoff of 3 μU/ml for the following three reasons: i) The main objective of this study was not to estimate prevalence of hypothyroidism but rather to compare the prevalence estimates across categories of predictor variables like waist circumference; ii) The criteria used for diagnosis of clinical thyroid states used in this study are based on clinical practice guidelines recommended by the American Association of Clinical Endocrinologists and the American Thyroid Association [34] and therefore follow the standards-of-care; and iii) Using these criteria, cross-ethnicity comparisons in American populations can be undertaken in future even if we have not included other ethnic groups in this study. In addition, we used TFI as a surrogate measure of thyroid function. This novel measure has the advantage that it combines the information content of TSH, FT3 and FT4 in the identification of hypothyroidism. However, we would like to point out that the clinical use of TFI has thus far not been validated in other populations and should be considered only illustrative rather than conclusive.

Biological plausibility in support of our findings is provided by a series of recent observations. First, thyroid stimulating hormone receptors (TSHR) are present in tissues other than the thyroid, especially in differentiating adipocytes [35]. Whilst it is conceivable that increasing TSHR concentration in obesity may attract additional release of TSH through a positive feedback to the pituitary, direct data in this regard are currently unavailable. As explained by Skudlinski et al. [36] and Mueller et al. [37], however the interactions between TSH and TSHR are very complex and therefore expectation of a simple positive feedback loop conjectured here may be overly simplistic. Second, thyroid hormone has been shown [38] to regulate expression of the gene encoding apolipoprotein M by binding to a hormone response element in the promoter of this gene. It is noteworthy that apolipoprotein M plays an important role in MS and obesity [38-40]. Third, in the 3 T3-L1 adipocytes it was observed [41] that *in vitro* treatment with triiodothyronine and thyroxine increased the expression of the gene encoding Plasminogen activator inhibitor 1, a key contributor in the inflammatory and cardiovascular complications of obesity. Fourth, triiodothyronine regulates the expression of the apolipoprotein AV gene, another important contributor to the complex pathogenesis of obesity and its complications [42]. Considered in totality, these studies lend biological credibility to our observation that WC is associated with thyroid function although the exact mechanism of this association remains unknown.

Our results are conceptually in agreement with the observed association of WC-based indices with altered thyroid function in other populations like Chinese children [43], Turkish women [44], other US ethnic groups [4], Italian euthyroid subjects [45] and Korean adults [46]. It is interesting that these studies have addressed different aspects of metabolic syndrome but none of these studies has directly and simultaneously assessed associations of the components of MS with thyroid dysfunction. For example, the study by Kitahara et al. [4], demonstrated that in a predominantly non-Hispanic white population of US men and women, higher values of BMI and WC were associated with increased TSH levels. However, a direct comparison of WC and BMI was not done in that study. Similarly, two studies [44,45] have specifically evaluated the association with a focus on insulin sensitivity and resistance with thyroid dysfunction. The study by Jung et al., used WHR as the index of central obesity and found similar results as those of ours but they did not include all the components included in this study. As a consequence of these differences a direct comparison of our results with other studies is not possible but all these studies support the paradigm that obesity and thyroid dysfunction are associated with each other.

Some limitations need to be considered before generalizing these results. First, both SAFHS and NHANES 2007–10 studies did not permit a direct investigation of a predictive value of future hypothyroidism – an important requirement before central obesity can be considered as a marker of thyroid dysfunction. Also, it has been argued that hypothyroidism itself may lead to obesity [47]. The direction of causal pathways cannot be established from this study. Second, we did not use the age-specific diagnostic criteria for defining hypothyroidism that are now in vogue [48]. However, since our results were adjusted for age, age^2^, sex and their interactions, we do not anticipate that our results would be influenced by any latent misclassification. Third, both SAFHS and NHANES 2007–10 datasets are, by design, cross-sectional in nature and follow-up data on these subjects is not available. Our reasoning that waist circumference might contribute to an increased risk of clinical hypothyroidism by predicting existing subclinical hypothyroidism is only a notional argument and can be affirmed only by longitudinal studies. Fourth, the reasons for some differences observed between males and females in the two SAFHS and NHANES 2007–10 datasets (for example, WC was significantly associated with TFI in females in SAFHS but in males in NHANES 2007–2010) are unclear and need to be investigated in future studies. It is possible that these significance values are primarily a result of the gender differential across the two datasets (SAFHS had 61% females while NHANES 2007–2010 had only 48% females). However, other reasons of biological of epidemiological dispositions cannot be overruled. Nevertheless, the fact that sex-adjusted results showed significant association of WC with TFI in both datasets demonstrates that the stratification due to sex may be minimal. Fifth, in our study we used the recommended [49] cutoffs for WC and BMI however there is now a growing opinion that “metabolically healthy obese” (MHO) subgroup [50] of MS is a special group with subclinical obesity or an overweight status that is associated with increased cardiovascular risks. Our study cannot directly comment on this but it is conceivable that this group may also be associated with altered thyroid disease risks. Future studies need to consider if central and general obesity are also associated with MHO status and therefore are able to identify individuals with increased likelihood of thyroid dysfunction.

Lastly, for dichotomizing the WC values we used cutoffs of ≥102 cm in men and ≥88 cm in women. While these cutoffs have been previously used for all US populations [49], lower cutoffs – especially those associated with increased likelihood of cardiovascular diseases – may provide even more sensitive prediction of thyroid dysfunction at the expense of specificity. The IDF recommends a cutoff of ≥90 cm and ≥80 cm for men and women of Mexican American and Hispanic origin [51]. As demonstrated by Ford et al. [52], these differences in the cutoffs can significantly influence the estimates of prevalence of abdominal obesity. At this time it is unclear how these definitions might impact the association of central obesity with thyroid dysfunction. Therefore, additional studies are required to investigate the potential effect of sliding cutoffs on the association of dichotomized WC with thyroid dysfunction.

Our study has an important indirect implication. Combined with the results of previous studies our results raise the possibility of considering central obesity as a screening adjunct to detect thyroid dysfunction. Recommendations for thyroid disease screening vary widely. The American Thyroid Association recommends [53] beginning screening at 35 y with 5-yearly follow-ups. The American College of Physicians recoomends screening in women over 50 who have at least one symptom suggestive of thyroid disease [54]. The American Academy of Family Physicians recommends [55] screening for high risk populations but the list of high-risk groups includes diabetes, autoimmune disorders, women with a history of thyroid disease, pregnant women and women over 35 years. The American Association of Clinical Endocrinologists recommends screening before childbearing age or pregnancy or during the first trimester [56]. Despite these varied recommendations the emerging common thread is that thyroid screening is likely to be beneficial in high-risk populations. In this vein, our observations raise the possibility that screening for thyroid dysfunction in Mexican Americans based on waist circumference, especially at younger ages, may yield additional benefits. Future studies need to carefully address this possibility.

## Conclusions

In two disparate and large population based studies of Mexican Americans, we found that WC is an independent predictor of thyroid function. This association is independent of age, gender and BMI. These results implore that in high-risk, high-prevalence populations like Mexican Americans, consideration needs to be given to WC as a potential screening tool for thyroid dysfunction Future studies need to evaluate the screening benefits, harms and costs associated with prediction of incident thyroid dysfunction based on current evidence of central obesity.

## Abbreviations

CDF: Cumulative density function; CO: Clinical hypothyroidism; CR: Clinical hyperthyroidism; FT3: Free triiodothyronine; FT4: Free thyroxine; HDL-C: High density lipoprotein cholesterol; MS: Metabolic syndrome; NHANES: National Health and Nutritiona Examination Survey; ROC: Receiver operating characteristic; RT3C: Reverse triiodothyronine; SAFHS: San Antonio Family Heart Study; SO: Subclinical hypothyroidism; SR: Subclinical hyperthyroidism; T2D: Type 2 diabetes; TBG: Thyroxine binding globulin; TFI: Thyroid function index; THG: Thyroglobulin; THRB: Thyroid hormone receptor beta; TSH: Thyroid stimulating hormone; TSHR: Thyroid stimulating hormone receptor; TT3: Total triiodothyronine; TT4: Total thyroxine; WC: Waist circumference; WHR: Waist-hip ratio.

## Competing interests

The authors declare that they have no competing interests.

## Authors’ contributions

MM and HK conceptualized the study, conducted the analyses and drafted the manuscript. JEC and JB shared the data, helped in conceptualizing the study, reviewed and wrote parts of the manuscript. PBS, AGC, RD, JB and JEC collected the thyroid function data. TDD, LA and MCM reviewed and wrote parts of the manuscript. All authors read and approved the final manuscript.

## Pre-publication history

The pre-publication history for this paper can be accessed here:

http://www.biomedcentral.com/1472-6823/14/46/prepub

## Supplementary Material

Additional file 1 This file contains three Supplementary Tables mentioned in the main text. Click here for file
