# Supplementary material for: Increased waist circumference is independently associated with hypothyroidism in Mexican Americans: replicative evidence from two large, population-based studies
Source: BMC Endocr Disord. 2014 Jun 10;14:46. doi: 10.1186/1472-6823-14-46 (PMC4057819; doi:10.1186/1472-6823-14-46)
Supplement: Additional file 1 — This file contains three Supplementary Tables mentioned in the main text. [file 1472-6823-14-46-S1.docx]

**Supplementary Table 1: Definitions for dichotomous traits used in the study**

| **Trait** | **Definition** |
| --- | --- |
| Central obesity | Waist circumference ≥102 cm in males and ≥88 cm in females.([1](#_ENREF_1)) |
| Raised triglycerides | Serum triglycerides ≥150 mg/dL (1.7 mmol/L) or receipt of lipid-lowering drugs.([2](#_ENREF_2)) |
| Low-HDL | Serum high density lipoprotein cholesterol concentration <40 mg/dL (1.03 mmol/L) in males, <50 mg/dL (1.29 mmol/L) in females or receipt of lipid-lowering drugs.([2](#_ENREF_2)) |
| Type 2 diabetes | American Diabetes Association criteria ([3](#_ENREF_3), [4](#_ENREF_4)) |
| High blood pressure | systolic blood pressure ≥130 or diastolic blood pressure ≥85 mm Hg or receipt of antihypertensive drugs.([2](#_ENREF_2)) |
| Metabolic syndrome | International Diabetes Federation ([2](#_ENREF_2)) definition: Presence of central obesity combined with any two of the following: raised triglycerides, low HDL-C, high blood pressure and raised fasting plasma glucose (≥5.6 mmol/L, previously diagnosed type 2 diabetes or receiving anti-diabetic medication) |

**References:**

1. **Alberti KG, Eckel RH, Grundy SM, Zimmet PZ, Cleeman JI, Donato KA, Fruchart JC, James WP, Loria CM, Smith SC, Jr.** 2009 Harmonizing the metabolic syndrome: a joint interim statement of the International Diabetes Federation Task Force on Epidemiology and Prevention; National Heart, Lung, and Blood Institute; American Heart Association; World Heart Federation; International Atherosclerosis Society; and International Association for the Study of Obesity. Circulation 120:1640-1645

2. **IDF** 2006 The IDF consensus worldwide definition of the metabolic syndrome. Brussels, Belgium: IDF Communications

3. **Genuth S, Alberti KG, Bennett P, Buse J, Defronzo R, Kahn R, Kitzmiller J, Knowler WC, Lebovitz H, Lernmark A, Nathan D, Palmer J, Rizza R, Saudek C, Shaw J, Steffes M, Stern M, Tuomilehto J, Zimmet P** 2003 Follow-up report on the diagnosis of diabetes mellitus. Diabetes care 26:3160-3167

4. **ADA** 2003 Report of the expert committee on the diagnosis and classification of diabetes mellitus. Diabetes care 26 Suppl 1:S5-20

**Supplementary Table 2. Direct comparison of the association of waist circumference and body mass index with thyroid function index using multivariate interactive model.**

| Subgroup | WC | | BMI | | WCxBMI | |
| --- | --- | --- | --- | --- | --- | --- |
|  | β | p | β | p | β | p |
| **SAFHS** | | | | | | |
| All | 0.2184 | 0.001 | -0.0389 | 0.538 | 0.0314 | 0.810 |
| Males | 0.4034 | 0.001 | -0.3018 | 0.011 | 0.0062 | 0.893 |
| Females | 0.1928 | 0.018 | 0.0012 | 0.989 | 0.0350 | 0.208 |
| **NHANES 2007-10** | | | | | | |
| All | 0.1311 | 0.066 | 0.0290 | 0.675 | 0.0335 | 0.218 |
| Males | 0.2797 | 0.004 | -0.0877 | 0.333 | 0.0425 | 0.069 |
| Females | 0.1929 | 0.070 | -0.0617 | 0.579 | 0.0087 | 0.819 |

**Supplementary Table 3. Direct comparison of the association of central obesity (WC ≥ 102 cm in males and ≥88 cm in females) and general obesity (BMI ≥ 30 Kg/m^2^) with thyroid function index using multivariate interactive model.**

| Subgroup | Central obesity | | General obesity | | Central obesity x  General obesity | |
| --- | --- | --- | --- | --- | --- | --- |
|  | β | p | β | p | β | p |
| **SAFHS** | | | | | | |
| All | 0.3601 | <0.001 | 0.1588 | 0.289 | -0.2087 | 0.243 |
| Males | 0.6208 | 0.011 | 0.1029 | 0.547 | -0.6087 | 0.048 |
| Females | 0.3312 | 0.004 | 0.3426 | 0.234 | -0.3165 | 0.303 |
| **NHANES 2007-10** | | | | | | |
| All | 0.2952 | 0.002 | -0.0230 | 0.925 | 0.0571 | 0.832 |
| Males | 0.2528 | 0.161 | 0.0095 | 0.970 | 0.1220 | 0.698 |
| Females | 0.2160 | 0.020 | 1.9148 | <0.001 | -1.9068 | <0.001 |
